# Critical Roles of the WASP N-Terminal Domain and Btk in LPS-Induced Inflammatory Response in Macrophages

**DOI:** 10.1371/journal.pone.0030351

**Published:** 2012-01-12

**Authors:** Chisato Sakuma, Mitsuru Sato, Takato Takenouchi, Joe Chiba, Hiroshi Kitani

**Affiliations:** 1 Animal Immune and Cell Biology Research Unit, National Institute of Agrobiological Sciences, Tsukuba, Ibaraki, Japan; 2 Department of Biological Science and Technology, Graduate School of Faculty of Industrial Science and Technology, Tokyo University of Science, Noda, Chiba, Japan; University of California Merced, United States of America

## Abstract

While Wiskott-Aldrich syndrome protein (WASP) plays critical roles in TCR signaling as an adaptor molecule, how it transduces innate immune signals remains to be elucidated. To investigate the roles of WASP in innate immune cells, we established bone marrow-derived macrophage (BMDM) cell lines from WASP15 transgenic (Tg) mice overexpressing the WASP N-terminal region (exons 1–5). Upon LPS stimulation, WASP15 Tg BMDM cell lines produce lower levels of inflammatory cytokines, such as TNF-α, IL-6, and IL-12p40 than the wild-type BMDM cell line. In addition, the production of nitric oxide by WASP15 Tg BMDM cells in response to LPS and IFN-γ was significantly impaired. Furthermore, we uncovered that the WASP N-terminal domain associates with the Src homology (SH) 3 domain of Bruton's tyrosine kinase (Btk). Overexpression of the WASP N-terminal domain diminishes the extent of tyrosine phosphorylation of endogenous WASP in WASP15 Tg BMDM cells, possibly by interfering with the specific binding between endogenous WASP and Btk during LPS signaling. These observations strongly suggest that the interaction between WASP N-terminal domain and Btk plays important roles in the LPS signaling cascade in innate immunity.

## Introduction

Wiskott-Aldrich syndrome (WAS) is an X-linked immunodeficiency characterized by eczema, thrombocytopenia, and susceptibility to infection, and accompanied by malignant lymphoma or serious autoimmune disease in severe cases. WAS results from various types of gene mutations in WAS protein (WASP) [1]. Symptoms of WAS are consistent with cytoskeletal defects in hematopoietic cells and suggest possible roles for WASP in actin-based processes [2]. In exploration of T cells from WAS patients and WASP-deficient mice, ectopic actin polymerization was observed at the immunological synapse after TCR ligation and resulted in impairment of IL-2 production upon TCR stimulation [3], [4]. WASP is also involved in the cytoskeletal rearrangement of innate immune cells. WASP-deficient monocytes and macrophages showed poor formation of the actin-rich phagocytic cup [5], and developed defects in polarization and migration in response to inflammatory chemokines *in vitro* [6], [7].

WASP contains several functional domains, including an N-terminal enabled/vasodilator-stimulated phosphoprotein (Ena/VASP) homology 1 (EVH1) domain (also known as WASP homology 1 (WH1) domain), a GTPase-binding domain (GBD), a proline-rich region (PRR), and a C-terminal verproline/cofilin/acidic (VCA) domain. The presence of multiple domains suggests that WASP recruits various kinds of adaptor molecules, protein tyrosine kinases, and actin-binding proteins, and therefore connects tyrosine kinase signaling to cellular motility devices driven by actin polymerization [8], [9].

Most gene mutations have been mapped to the WASP N-terminal region including the EVH1 domain [10], suggesting that this domain is important for WASP function. However, how the dysfunction of this domain is related to the WAS disease phenotype remains unknown. To clarify the function of the WASP N-terminal domain in immune response, we have developed transgenic (Tg) mice that overexpress WASP exons 1–5 (aa 1–171, designated WASP15) [11]. T cells from WASP15 Tg mice were impaired in proliferative response, as well as in TCR-induced IL-2 production. Additionally, microglia from WASP15 Tg mouse brains were impaired in the LPS-induced production of inflammatory cytokines [12]. These findings suggest that the WASP N-terminal domain plays critical roles in the adaptive immune response, as well as in the innate immune signal cascades.

To further investigate the possible functions of WASP in innate immune cells, we established bone marrow-derived macrophage (BMDM) cell lines from WASP15 Tg mice and the immune response to stimulation with LPS was compared to the response of wild-type BMDM cells. We also identified the Src homology (SH) 3 domain of Bruton's tyrosine kinase (Btk) as a binding counterpart of the WASP N-terminal domain using the *in vitro* binding assay. Overexpression of the WASP N-terminal domain in BMDM cells in WASP15 Tg mice interferes with the interaction between endogenous WASP and Btk, resulting in an impaired inflammatory response to LPS.

## Results

### Establishment of BMDM cell lines from wild-type and WASP15 Tg mice

**Figure 1:**
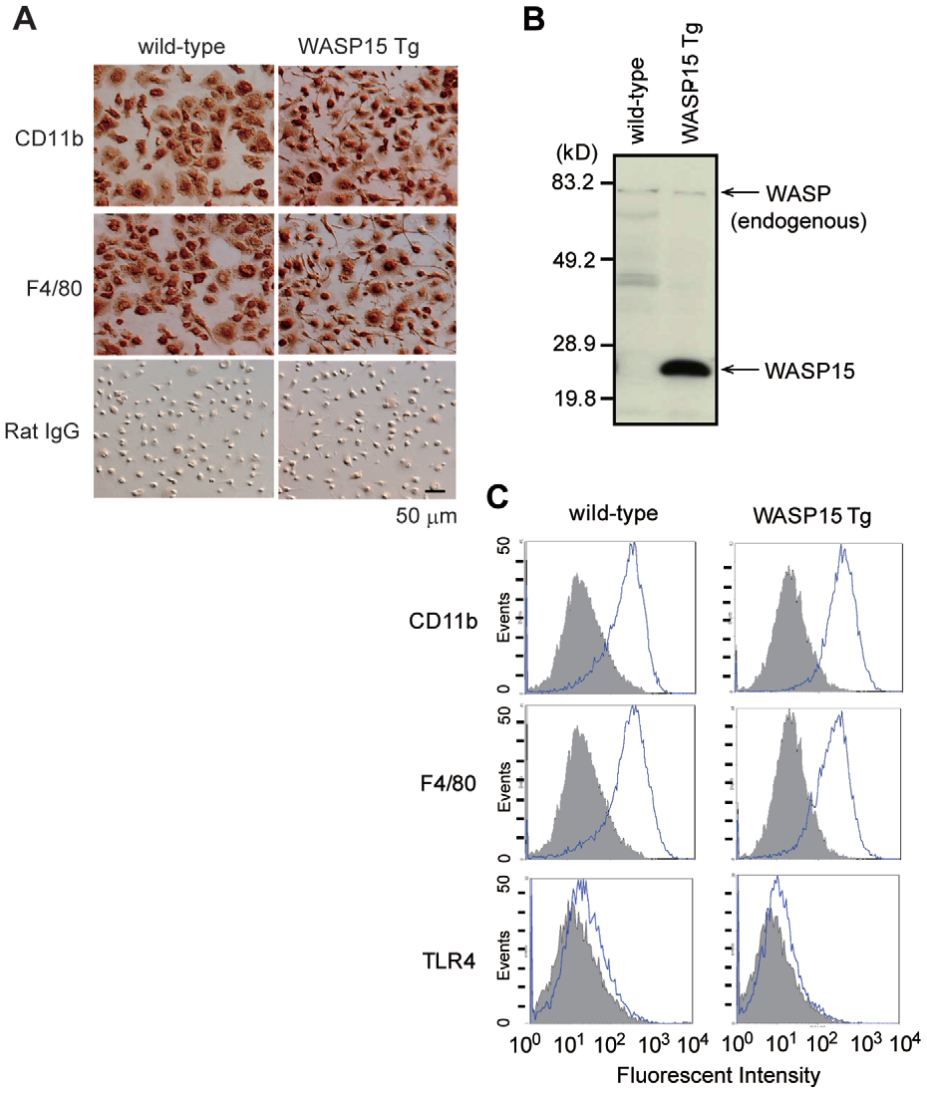
Establishment of bone marrow-derived macrophage (BMDM) cell lines from wild-type and WASP15 Tg mice. BMDMs were prepared from primary cultures of bone marrow with a human c-*myc* gene-containing retroviral vector. (A) BMDM cell lines were immunocytochemically stained with anti-CD11b and anti-F4/80 antibodies, but not with control Rat IgG. Bar = 50 µm (B) Expression of truncated WASP and endogenous WASP in BMDMs. Cell lysates were analyzed by Western blotting with an anti-WASP mAb. (C) FACS analysis of wild-type and WASP15 Tg BMDM cell lines. Cells were stained with PE-conjugated anti-CD11b, anti-F4/80 and anti-TLR4 antibodies (open histogram), or isotype-matched control Ab (filled histogram). All results are representative of three independent experiments.

Bone marrow-derived macrophages prepared from wild-type and WASP15 Tg mice were infected with a c-*myc* gene-containing retroviral vector, and representative BMDM clonal cell lines were established. Both wild-type and WASP15 Tg BMDM cell lines were strongly immunostained with rat monoclonal antibodies against mouse macrophages, such as CD11b or F4/80 (Fig. 1A). In contrast, control rat IgG did not show positive staining in these cell lines. Morphological and immunohistochemical observations strongly suggest that these immortalized cell lines were derived from BMDM.

The expression of the c-*myc* gene in wild-type and WASP15 Tg BMDMs was confirmed by RT-PCR (data not shown). In addition, Western blot analysis showed that the truncated WASP (WASP15) was strongly expressed only in WASP15 Tg BMDM, while endogenous WASP was equivalently expressed in BMDMs (Fig. 1B).

### Expression of TLR4 in BMDMs

TLR4, a receptor for LPS [13], is highly expressed in macrophages and transduces inflammatory signaling, such as production of inflammatory cytokines [14]. To compare the expression levels of TLR4 between wild-type and WASP15 Tg BMDMs, we performed a FACS analysis with anti-TLR4 antibody. There was no significant difference in the expression levels of TLR4, CD11b, or F4/80 between wild-type and WASP15 Tg BMDMs (Fig. 1C). These findings suggest that the overexpression of WASP15 does not affect the expression of macrophage cell surface molecules such as CD11b, F4/80, and TLR4, in BMDM.

### Impairment of cytokine production in WASP15 Tg BMDM upon LPS stimulation

**Figure 2:**
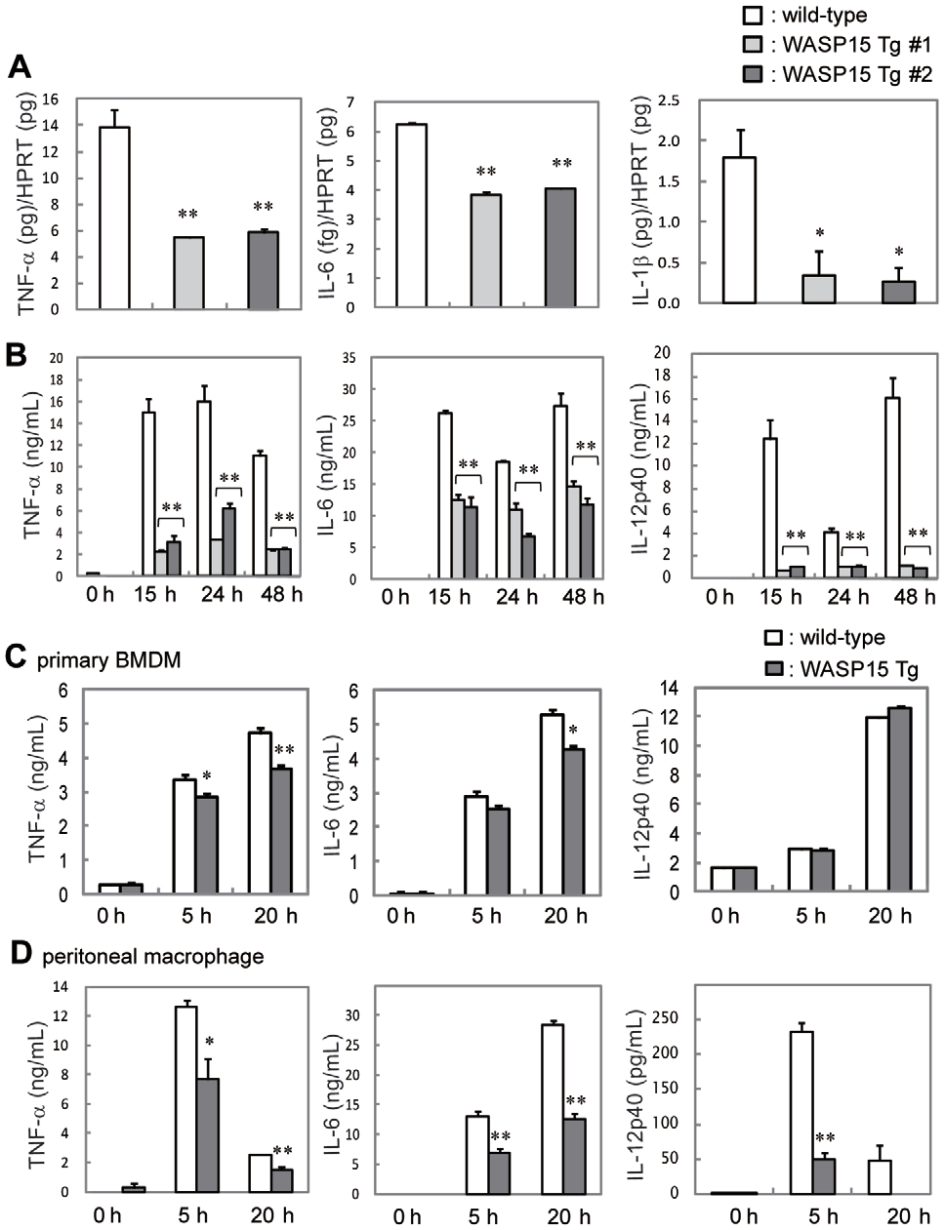
Cytokine production induced by LPS stimulation in BMDM cell lines. (A) Quantitative real-time PCR was performed on RNA derived from wild-type and WASP15 Tg BMDMs following LPS stimulation. Expression levels are reported as transcripts of TNF-α, IL-6, and IL-1β per transcript of control HPRT. WASP15 Tg BMDM clones #1 and #2 were independently isolated. (B) Wild-type and WASP15 Tg BMDMs were cultured in medium in either the presence or the absence of LPS. Each cell culture supernatant was collected at 0, 15, 24, and 48 h after stimulation. (C) Primary cultured BMDMs and (D) peritoneal macrophages from wild-type and WASP15 Tg mice were cultured in medium in either the presence or the absence of LPS. Each cell culture supernatant was collected at 0, 5, and 20 h after stimulation. Concentrations of TNF-α, IL-6 and IL-12p40 in the culture supernatant were quantified by ELISA. Values represent means ± SEs of triplicate cultures. The profiles are typical examples of at least three independent experiments. A significant difference is indicated by *(p<0.01) and **(p<0.001).

Macrophages activated by LPS secrete a wide variety of inflammatory cytokines [13], [14]. To assess the effects of overexpression of WASP15 in the LPS signaling pathway, quantitative real-time PCR was performed on RNA isolated from wild-type and WASP15 Tg BMDMs (clones #1 and #2) activated by LPS stimulation. In contrast to the marked upregulation of TNF-α, IL-6 and IL-1β gene transcription upon LPS stimulation in wild-type BMDMs, WASP15 Tg BMDMs showed one-third of the levels of TNF-α and IL-1β and two-thirds of the levels of IL-6 transcription (Fig. 2A). Furthermore, ELISA analysis demonstrated that the levels of secreted inflammatory cytokines, such as TNF-α, IL-6, and IL-12p40, upon LPS stimulation were significantly impaired in WASP15 Tg BMDMs (Fig. 2B). To confirm the impairment of inflammatory cytokine production upon LPS stimulation, ELISA analyses were performed in primary cultured BMDM cells and peritoneal macrophages from wild-type and WASP15 Tg mice. In primary WASP15 Tg BMDMs, production of TNF-α and IL-6 was marginally reduced. In addition, the level of IL-12p40 production was similar to wild-type primary BMDMs (Fig. 2C). In this assay, the primary cultured BMDMs were pre-stimulated with M-CSF before LPS stimulation, and this might have affected the inflammatory cytokine responses in primary cultured BMDMs. On the other hand, impairment of TNF-α, IL-6 and IL-12p40 production upon LPS stimulation was confirmed in primary cultured peritoneal macrophages from WASP15 Tg mice (Fig. 2D). These results suggest that the WASP N-terminal region is important for WASP function in inflammatory cytokine production following LPS stimulation in both BMDM and peritoneal macrophages.

### Impairment of NO production in WASP15 Tg BMDM upon LPS and IFN-γ stimulation

**Figure 3:**
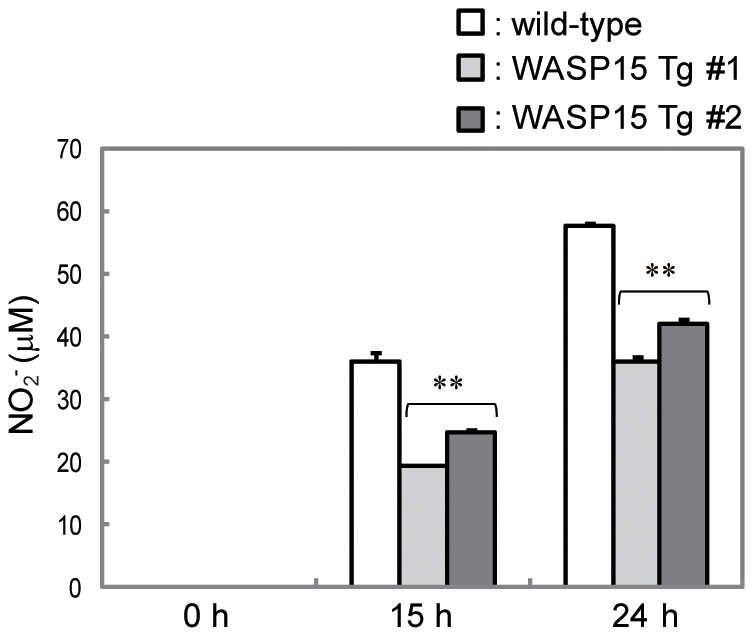
NO production induced by LPS and IFN-γ stimulation in BMDMs. Wild-type and WASP15 Tg BMDMs were cultured in medium alone or in combination with LPS and IFN-γ. Each culture supernatant was collected at 15 and 24 h after stimulation. NO_2_^−^ in the culture supernatant was quantified using Griess reagent. WASP15 Tg BMDM clones #1 and #2 were independently isolated. Values represent means ± SEs of triplicate cultures. Profiles are representative of three independent experiments. A significant difference is indicated by **(p<0.001).

Macrophages activated with LPS and IFN-γ produce bactericidal substances, such as NO [15], [16]. To assess the effect of WASP15 overexpression on the NO production pathway in macrophages, wild-type and WASP15 Tg BMDMs (clones #1 and #2) were stimulated by combination with LPS and IFN-γ and the NO_2_^−^ secreted in the culture medium was quantified using Griess reagent. After 24 h of stimulation, wild-type BMDM produced approximately 60 µM of NO_2_^−^. In contrast, WASP15 Tg BMDM produced 30–40 µM of NO_2_^−^ (Fig. 3). By stimulation with either LPS or IFN-γ alone, the secretion of NO_2_^−^ from wild-type and WASP15 Tg BMDMs was not observed in the assay (data not shown). These results suggest that the overexpressed WASP15 may inhibit some critical steps in the NO synthesis pathway induced by LPS and IFN-γ in macrophages.

### Activation of NF-κB and MAPK pathways induced by LPS stimulation in BMDMs

**Figure 4:**
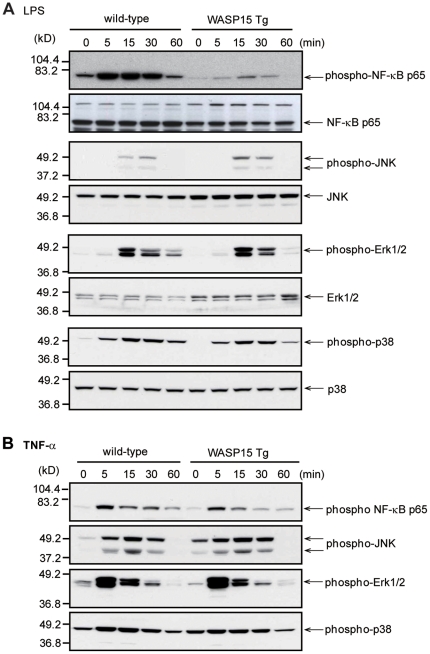
Activation of NF-κB and MAPKs induced by LPS or TNF-α in BMDMs. Wild-type and WASP15 Tg (#1) BMDMs were stimulated with (A) LPS or (B) TNF-α for the time indicated and lysed. Proteins from cellular lysates were separated by SDS-PAGE and immunoblotted with anti-phospho-specific antibodies to NF-κB, JNK, Erk1/2, and p38. Anti-NF-κB, JNK, Erk1/2, and p38 antibodies were used to show equal protein loading. The immunoblots are representative of three independent experiments.

The activation of NF-κB is essential for inflammatory cytokine production in activated macrophages [14]. To assess whether overexpression of the WASP N-terminal domain affects the LPS-induced NF-κB signaling pathway in macrophages, the extent of LPS-induced phosphorylation of NF-κB was compared between wild-type and WASP15 Tg BMDMs using Western blot analysis. In wild-type BMDMs, phosphorylation of NF-κB p65 (Ser-536) was rapidly induced and maintained higher levels after 30 min of LPS stimulation. In contrast, phosphorylation of NF-κB p65 in WASP15 Tg BMDMs was maintained at lower levels during the experiments (Fig. 4A). Levels of total NF-κB p65 protein were comparable between wild-type and WASP15 Tg BMDMs (Fig. 4A). However, phosphorylation profiles of MAPKs such as JNK, Erk1/2, and p38 MAPK upon LPS stimulation were similar between wild-type and WASP15 Tg BMDMs (Fig. 4A), suggesting that overexpression of WASP15 affects the activation of NF-κB, but not MAPKs, upon LPS stimulation in BMDMs.

As a positive control, BMDMs were stimulated with TNF-α *in vitro*, and then the extent of phosphorylation of NF-κB and MAPKs was analyzed by Western blot analysis. The phosphorylation profiles for NF-κB and MAPKs upon TNF-α stimulation were similar between wild-type and WASP15 Tg BMDMs (Fig. 4B). These findings strongly suggest that the overexpressed WASP15 specifically blocks the phosphorylation of NF-κB in the LPS signaling cascade, but does not affect the phosphorylation of NF-κB in the TNF-α signaling cascade in macrophages.

### Specific interaction between the WASP N-terminal domain and the SH3 domain of Btk

**Figure 5:**
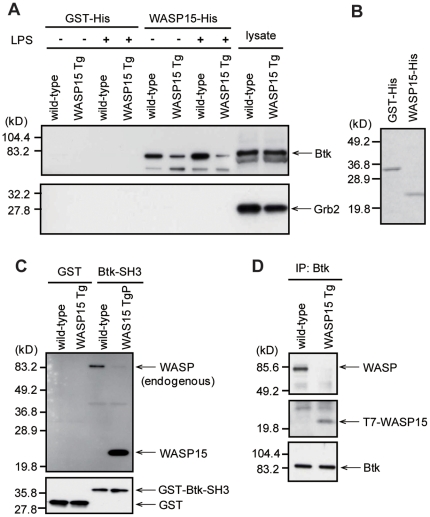
Inhibition of WASP-Btk interactions by overexpression of the WASP N-terminal domain. (A) Wild-type and WASP15 Tg BMDMs (clone #1) without (−) or with (+) LPS stimulation, were lysed with RIPA buffer, incubated with GST-His or WASP15-His fusion proteins and immunoprecipitated with anti-His tag Ab. Immunocomplexes were analyzed by Western blot with anti-Btk Ab or anti-Grb2 Ab. (B) Equal amounts of GST-His and WASP15-His fusion proteins, isolated using a pull-down assay, were separated by SDS-PAGE and stained with Coomassie blue. (C) Wild-type and WASP15 Tg BMDMs (clone #1) were lysed and incubated with GST or GST-Btk-SH3 fusion protein non-covalently bound to glutathione sepharose beads. Bound proteins were analyzed by Western blotting with anti-WASP mAb or anti-GST pAb. (D) Wild-type and WASP15 Tg BMDMs (clone #1) were lysed and immunoprecipitated with anti-Btk mAb. Immunocomplexes were analyzed by Western blotting with anti-WASP pAb, anti-T7-tag pAb, and anti-Btk pAb. The immunoblots are representative of three independent experiments.

Recently, we demonstrated that the WASP N-terminal domain specifically binds to the SH3 domain of Fyn tyrosine kinase in TCR signaling, and the interaction between endogenous WASP and Fyn was strongly inhibited by overexpression of the WASP N-terminal domain in WASP15 Tg T cells [17]. Several lines of evidence suggest that Btk is involved in TLR4 signal transduction in macrophages [18], [19]. Therefore, Btk may be a binding partner of the WASP N-terminal domain in LPS-activated macrophages. To confirm this possibility, an *in vitro* binding assay was performed using the WASP15-His pull-down assay. The WASP15-His strongly bound to Btk in wild-type BMDMs regardless of LPS stimulation. In contrast, the specific interaction between WASP15-His and Btk was inhibited in WASP15 Tg BMDMs (Fig. 5A, upper panel). Growth factor receptor-bound protein 2 (Grb2) contains two SH3 domains, but the interaction between WASP15-His and Grb2 was not detected in wild-type and WASP15 Tg BMDMs (Fig. 5A, lower panel). The protein levels of GST-His and WASP15-His used in this assay were comparable (Fig. 5B). The protein levels of endogenous Btk and Grb2 in these BMDMs were comparable (Fig. 5A).

A reciprocal binding assay using the GST or GST-Btk-SH3 fusion proteins confirmed the strong binding of WASP to the GST-Btk-SH3 in wild-type BMDMs, while this interaction was greatly inhibited in WASP15 Tg BMDMs (Fig. 5C), which overexpress the WASP N-terminal domain. The protein levels of GST and GST-Btk-SH3 were comparable (Fig. 5C). These results strongly suggest that overexpression of WASP15 specifically interferes with the specific binding between the WASP N-terminal domain and the SH3 domain of Btk, which mediates the LPS-signaling in macrophages.

To confirm the binding of endogenous WASP and Btk in BMDMs, wild-type and WASP15 Tg BMDM cell lysates were immunoprecipitated with anti-Btk mAb, and immunocomplexes were immunoblotted with anti-WASP pAb. In the immunoprecipitation analysis, specific interaction between endogenous WASP and Btk was clearly detected in wild-type BMDMs (Fig. 5D, upper panel). In contrast, the interaction between WASP and Btk was severely inhibited in WASP15 Tg BMDMs (Fig. 5D, upper panel). In addition, the competitive binding of the truncated WASP to Btk was demonstrated by immunoblotting with anti-T7 tag Ab (Fig. 5D, center panel). Btk was equivalently immunoprecipitated in both BMDMs (Fig. 5D, lower panel). Taken together, these observations strongly suggest that the interaction between endogenous WASP and Btk in macrophages has critical roles in inflammatory responses to LPS in macrophages, possibly mediated by the binding of the Btk-SH3 and the WASP N-terminal domains.

### Formation of WIP-WASP-Btk complex in BMDMs

**Figure 6:**
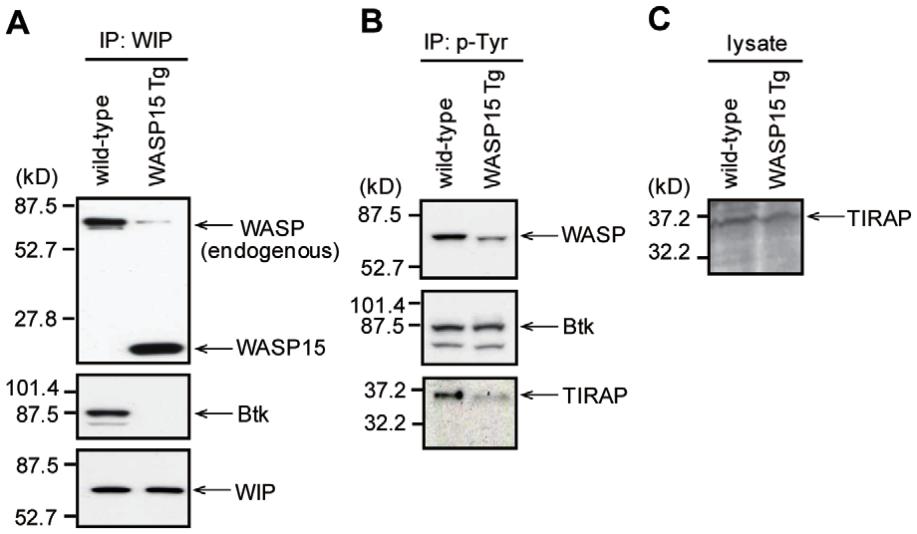
Complex formation of WIP-WASP-Btk and phosphorylation of WASP and TIRAP upon LPS stimulation in BMDMs. (A) Wild-type and WASP15 Tg BMDMs (clone #1) were lysed and immunoprecipitated with anti-WIP pAb. Immunocomplexes were analyzed by Western blotting with anti-WASP mAb, anti-Btk mAb, and anti-WIP mAb. (B) LPS-stimulated wild-type and WASP15 Tg BMDMs (clone #1) were lysed and immunoprecipitated with anti-phosphotyrosine (p-Tyr) mAb. Immunocomplexes were analyzed by Western blotting with anti-WASP pAb, anti-Btk pAb, or anti-TIRAP pAb. The immunoblots are representative of three independent experiments. (C) Equivalent expression of TIRAP in wild-type and WASP15 Tg BMDMs (clone #1) was demonstrated by Western blotting with an anti-TIRAP pAb. The immunoblots are representative of three independent experiments.

WASP-interacting protein (WIP) is known to bind to the WASP N-terminal EVH1 domain [20]–[22]. Previously, we demonstrated that the WASP N-terminal domain binds to both Fyn-SH3 and WIP in T cells and these three molecules associate closely in the complex to modulate transcriptional activation of IL-2 in response to TCR signaling [17]. To confirm the possible roles of WIP-WASP-Btk complex in macrophages, wild-type and WASP15 Tg BMDM cell lysates were immunoprecipitated with anti-WIP pAb, and immunocomplexes were immunoblotted with anti-WASP mAb and anti-Btk mAb. In the immunoprecipitation analysis, interaction between endogenous WIP and WASP was detected in wild-type BMDMs (Fig. 6A, upper panel). In contrast, the interaction between WIP and WASP was strongly inhibited in WASP15 Tg BMDMs (Fig. 6A, upper panel), possibly by the competitive binding of WASP15 to WIP. Furthermore, the interaction of Btk was clearly detected in wild-type BMDMs; however, WASP15 overexpression inhibited the binding of Btk in WASP15 Tg BMDMs (Fig. 6A, center panel). Although WASP N-terminal domain bound to both Btk and WIP, we could not detect the Btk-WASP15-WIP complex in the immunoprecipitation analysis (Fig. 6A, center panel). One possible reason is that the truncated WASP15 cannot form the proper complex of Btk and WIP in BMDM. WIP was equivalently immunoprecipitated in both BMDMs (Fig. 6A, lower panel). Taken together, these observations strongly suggest that the WASP N-terminal domain has an affinity for both Btk and WIP, and the formation of the WIP-WASP-Btk complex is critical to LPS signaling in macrophages.

### Impairment of LPS-induced tyrosine phosphorylation of WASP and TIRAP in WASP15 Tg BMDMs

To assess whether overexpression of the WASP N-terminal domain affects tyrosine phosphorylation of WASP induced by LPS stimulation, the extent of LPS-induced tyrosine phosphorylation of WASP was compared between wild-type and WASP15 Tg BMDMs using immunoprecipitation analysis. Tyrosine phosphorylation of WASP upon LPS stimulation was clearly detected in wild-type BMDMs, but was markedly reduced in WASP15 Tg BMDMs (Fig. 6B, upper panel). In contrast, Btk was equivalently tyrosine phosphorylated upon LPS stimulation in these BMDMs (Fig. 6B, center panel). These results suggest that overexpression of the WASP N-terminal domain inhibits tyrosine phosphorylation of WASP by interfering in the Btk binding to the WASP N-terminus, but does not affect the activation of Btk upon LPS stimulation.

Toll/IL-1 receptor domain-containing adapter protein (TIRAP), also known as MyD88 adapter-like protein (Mal), acts as a bridging adaptor for TLR4 and MyD88 [23], [24]. TIRAP is tyrosine phosphorylated by Btk following activation of TLR4, leading ultimately to NF-κB activation [19], [23]. To assess whether overexpression of the WASP N-terminal domain affects tyrosine phosphorylation of TIRAP induced by LPS stimulation, the extent of LPS-induced tyrosine phosphorylation of TIRAP was compared between wild-type and WASP15 Tg BMDMs. Tyrosine phosphorylation of TIRAP upon LPS stimulation was clearly detected in wild-type BMDMs, but was markedly reduced in WASP15 Tg BMDMs (Fig. 6B, lower panel), suggesting that the impairment of complex formation of WIP-WASP-Btk is reflected in the reduction of LPS-induced tyrosine phosphorylation of TIRAP in WASP15 Tg BMDMs. TIRAP was equivalently expressed in both BMDMs (Fig. 6C). Taken together, these observations strongly suggest that Btk, WASP, and WIP are closely associated in the complex to modulate inflammatory signals through LPS-activated TLR4 in macrophages.

## Discussion

This study has demonstrated that BMDM cell lines established from Tg mice that overexpress the WASP N-terminal domain exhibit impaired immunological responses to LPS, such as the production of various inflammatory cytokines or nitric oxide. Possible roles for WASP in innate immune signaling have been suggested in microglia [12]. WASP-overexpressing Tg microglia demonstrated impaired production of inflammatory cytokines upon LPS stimulation [12]. Additionally, LPS-induced phosphorylation of the transcription factor NF-κB was reduced in Tg microglia. The phosphorylation of the p65 subunit of NF-κB at Ser-536 is essential for the nuclear translocation and transcriptional activation of inflammatory cytokine genes [25], [26]. In wild-type BMDMs, phosphorylation of NF-κB p65 (Ser-536) was rapidly induced and maintained at higher levels after 30 min of LPS stimulation, while LPS-induced phosphorylation of NF-κB p65 was greatly inhibited in WASP15 Tg BMDMs (Fig. 4A), suggesting that the activation of NF-κB was impaired. In contrast, the activation profiles of MAPKs induced by LPS stimulation in WASP15 Tg BMDMs were indistinguishable from those of wild-type BMDMs. These findings confirm the important roles of WASP in the LPS signaling cascade in macrophage-lineage cells, which results in the production of NO and inflammatory cytokines through NF-κB activation.

Recently, Btk has been demonstrated to have important roles in LPS-TLR4 signaling in monocytic cells and macrophages. Doyle et al. demonstrated that Btk induces the phosphorylation of NF-κB p65 on Ser-536 in response to LPS [27], and then the expression of inflammatory cytokines are upregulated [25], [26]. In this study, we demonstrated that NF-κB p65 is highly phosphorylated at Ser-536 upon LPS stimulation in wild-type BMDM, while that of WASP15 Tg BMDM is markedly reduced (Fig. 4A), suggesting that impaired production of inflammatory cytokine in WASP15 Tg BMDM was caused by inactivation of NF-κB due to the competitive binding of WASP15 to Btk (Fig. 5C, D). However, Horwood et al. showed that Btk does not control the synthesis of IL-6 and IL-8 [28]. Their work particularly demonstrated that Btk lies within a p38 MAPK-dependent pathway that stabilizes TNF-α mRNA, but not IL-6 in human monocytes. In our study, LPS-induced phosphorylation of p38 MAPK was not affected by the overexpression of WASP N-terminal domain in WASP15 Tg BMDMs (Fig. 4A), but gene transcription of TNF-α, IL-6, and IL-1β was significantly reduced (Fig. 2A). These findings suggest that signaling pathway under LPS-TLR4 may be complicated, and further studies should be necessary for clarification of the mechanisms of LPS-induced immune response in macrophage.

TIRAP is a bridging adapter for TLR4 and MyD88, which is the key signaling molecule for TLR2 and TLR4 [24]. Gray et al. demonstrated that TIRAP can be tyrosine phosphorylated by Btk following activation of TLR2 and TLR4 [19]. This tyrosine phosphorylation of TIRAP by Btk is required for the activation of a pathway culminating in transactivation by the NF-κB p65 subunit [27], [29]. In the present study, the substantial tyrosine phosphorylation of TIRAP induced by LPS stimulation was observed in wild-type BMDMs, but not in WASP15 Tg BMDMs (Fig. 6B, lower panel), suggesting that the specific association of WASP with Btk is important for the tyrosine phosphorylation of TIRAP upon LPS stimulation in macrophages.

Btk is a non-receptor tyrosine kinase and belongs to the Tec family of kinases. Btk has several domains, including (from the N-terminus): the pleckstrin homology (PH) domain, Tec homology (TH) domain, SH2 domain, SH3 domain and kinase domain [30], [31]. The SH3 domain of Btk binds a variety of poly-proline-containing proteins, which have specific intracellular signaling roles [32]–[34]. The multiplicity of signals is guaranteed by the binding specificities of the domains of Btk to other signaling molecules. Although several lines of evidence suggest that Btk and WASP interact with each other through the SH3 and C-terminal PRR, respectively [35], [36], we demonstrated that the Btk SH3 domain specifically binds to the WASP N-terminal domain. This physiological interaction is inhibited by competitive binding of the overexpressed WASP N-terminal domain, resulting in the reduction of tyrosine phosphorylation of WASP upon LPS stimulation in WASP15 Tg BMDMs (Figs. 5D and 6B). These observations strongly support the hypothesis that Btk specifically interacts with the WASP N-terminal region through its SH3 domain and phosphorylates WASP to modulate WASP function in LPS-induced inflammatory signaling.

WIP is well known as a binding partner of WASP and its binding site has already been determined within the N-terminal 170 amino acids of WASP [37]. As shown in Btk-WASP binding, the interaction between endogenous WASP and WIP was inhibited by the competitive binding of the overexpressed WASP N-terminal domain in WASP15 Tg BMDMs (Fig. 6A, upper panel). Furthermore, WIP-WASP-Btk signaling complex was observed in wild-type BMDMs, while this complex formation was impaired in WASP15 Tg BMDMs (Fig. 6A).

WIP shuttles Fyn-associated WASP to the plasma membrane in T cells activated by TCR ligation [17], [21]. In macrophages, WIP closely associates with WASP and Btk through the N-terminal domain of WASP, and may shuttle them to the plasma membrane, where the TLR4 signal complex is localized. However, overexpressed WASP N-terminal domain competitively binds to Btk and WIP, and interferes the formation of the Btk-WASP-WIP complex in WASP15 BMDM. Under this condition, Btk cannot be recruited to the TLR4 signaling complex, where TIRAP is localized. Then Btk cannot phosphorylate tyrosine residue within TIRAP following LPS stimulation._These results strongly suggest that formation of the WIP-WASP-Btk complex is necessary for the activation of TLR4 signal complex upon LPS stimulation.

The phosphorylated tyrosine residue and C-terminal PRR of WASP may be targeted by SH2 or SH3 domain-containing molecules, which are involved in LPS-activated signaling events, including the tyrosine phosphorylation of TIRAP. The identification of molecules downstream of the WIP-WASP-Btk complex in the LPS signaling cascade will provide insight into the molecular mechanism underlying the inflammatory response in macrophages.

In conclusion, our experiments suggest that the WASP N-terminal domain binds to the SH3 domain of Btk, and this interaction plays an important role in modulating the inflammatory response in LPS-activated macrophages. Although the details of signaling through TLRs are currently being investigated, the identification of novel signaling molecules and their interactions during LPS-TLR4 signal transduction will help build alternative therapeutic strategies for septic shock and other inflammatory diseases.

## Materials and Methods

### Establishment of bone marrow-derived macrophage (BMDM) cell lines

BMDMs were cultured from the bone marrow of C57BL/6 mice and WASP15 Tg mice [11], according to a protocol described previously [38]. Primary BMDM cultures were infected with a replication-deficient retroviral vector containing the human c-*myc* and neomycin resistance genes (a gift from M. Noda, Kyoto University, Japan). After selection with medium containing G418 at 600 µg/mL, several clones were isolated by a limiting dilution method. BMDM cell lines were routinely cultured at 37°C in humidified 5% CO_2_/95% air with modifiedMEM containing 10% FCS supplemented with 100 µg/mL streptomycin, 100 U/mL penicillin and 4 mM L-glutamine.

### Immunocytochemistry

BMDMs seeded on eight-well chamber slides (5×10^4^ cells/well) were fixed with 10% formalin in PBS for 30 min at 4°C. After fixation, cells were washed with cold PBS, then incubated with 1% Triton X in PBS for 30 min at 4°C. Cells were washed with cold PBS and incubated with peroxidase blocking reagent (Dakocytomation, Glostrup, Denmark) for 10 min at 25°C to block endogenous peroxidase activity, followed by blocking with 5% normal goat serum and 1% BSA in PBS for 15 min at 25°C. Next, the primary antibodies against CD11b, F4/80, or control rat IgG (Serotec, Oxford, UK) were applied for 1 h at 25°C. The secondary incubations were performed with HRP-conjugated anti-rat IgG (Dakocytomation) for 1 h at 25°C. Finally, a colorimetric substrate, 3,3′-diaminobenzidine tetrahydrochloride (DAB) [EnVision™ kits/HRP (DAB), Dakocytomation], was applied according to the manufacturer's instructions. After additional washing with distilled water, the slides were dehydrated and mounted with coverslips using Mount-Quick (Daido Sangyo Co. Ltd, Japan).

### FACS analysis

BMDMs (5×10^5^ cells) were incubated with 10 µg/mL Fc-block (anti-CD16/32 monoclonal Ab (mAb); BD Pharmingen, San Diego, CA, USA) for 10 min at 4°C and stained with PE-conjugated anti-CD11b Ab (BioLegend, San Diego, CA, USA), anti-F4/80 Ab (Serotec), anti-TLR4 Ab (BD Pharmingen), or the isotype control Ab (Immunotech, Marseille, France) for 60 min at 4°C. After washing with PBS, cells were analyzed by flow cytometry (Bechman Coulter, EPICS XL).

### Quantitative real-time PCR

BMDMs were seeded in 100 mm petri dishes (1×10^6^ cells/dish) and treated with LPS (1 µg/mL; ultra pure *E. coli* 0111: B4 LPS, InvivoGen, San Diego, CA, USA) for 5 h at 37°C. RNA from the BMDMs was isolated using the SV RNA Isolation System (Promega, Madison, WI, USA). cDNA was obtained using the ReverTra Ace-α-® first-strand cDNA synthesis kit (Toyobo, Osaka, Japan) according to the manufacturer's instructions.

Real-time PCR for mouse inflammatory cytokines was performed in a LightCycler 1.5 (Roche Diagnostics, Basel, Switzerland). cDNA was amplified with the LightCycler TaqMan Master kit (Roche) using Universal ProbeLibrary probe #78 (Roche) and specific primer sets for mouse TNF-α, IL-6, IL-1β (Table 1). HPRT was employed as a standard, using Universal ProbeLibrary probe #22 (Roche) and a specific primer set (Table 1).

**Table 1:** Specific oligonucleotide primers used for PCR amplification.

TNF-α	Forward	5′-CTGTAGCCCACGTCGTAGC-3′
	Reverse	5′-TTGAGATCCATGCCGTTG-3′
IL-6	Forward	5′-TCTAATTCATATCTTCAACCAAGAGG-3′
	Reverse	5′-TGGTCCTTAGCCACTCCTTC-3′
IL-1β	Forward	5′-TGTAATGAAAGACGGCACACC-3′
	Reverse	5′-TCTTCTTTGGGTATTGCTTGG-3′
HPRT	Forward	5′-TGATAGATCCATTCCTATGACTGTAGA-3′
	Reverse	5′-AAGACATTCTTTCCAGTTAAAGTTGAG-3′

### Cytokine ELISA

BMDMs were cultured in 48-well plates (1×10^5^ cells/500 µL/well) with medium, in either the presence or the absence of LPS (10 µg/mL). The cell culture supernatant was collected at 15, 24, and 48 h after stimulation. Primary peritoneal macrophages were prepared from mice by peritoneal washing with cold PBS; similarly, they were treated with LPS and the culture supernatant was collected at 5 and 20 h after stimulation. The levels of TNF-α, IL-6, and IL-12p40 in the culture supernatant were quantified in triplicate with the ELISA MAX™ Set Deluxe (BioLegend) according to the manufacturer's instructions.

### NO assay

BMDMs were seeded in 48 well plates (1×10^5^ cells/500 µL/well) with medium in either the presence or the absence of LPS (10 µg/mL) plus IFN-γ (100 U/mL; PBL Biomedical Laboratories, Piscataway, NJ, USA). The cell culture supernatant was collected at 15 and 24 h after stimulation. The concentration of nitrite (NO_2_^−^) in the culture supernatant was quantified in triplicate using the Griess Reagent System (Promega) according to the manufacturer's instructions.

### Western blot analysis for activation of MAPKs and NF-κB

BMDMs were activated with either LPS (10 µg/ml) or recombinant mouse TNF-α (10 ng/mL; Roche) for different time intervals at 37°C. The activated cells were washed with PBS and lysed with SDS-sample buffer at 25°C. The cell lysates were separated by 12.5% SDS-PAGE and transferred to a polyvinlidene difluoride membrane (Bio-Rad, Hercules, CA, USA). The membrane was blocked with TBST buffer (10 mM Tris-HCl, pH 8.0, 0.15 M NaCl, and 0.05% Tween 20) containing 5% (w/v) non-fat dry milk. Blots were probed with anti-phospho-NF-κB p65 (Ser-536), anti-NF-κB p65, anti-phospho-JNK, anti-JNK, anti-phospho-Erk 1/2, anti-Erk 1/2, anti-phospho-p38, or anti-p38 antibodies (Cell Signaling Technology, Danvers, MA, USA), followed by HRP-conjugated anti-rabbit IgG (Dakocytomation). Immunoreactive proteins were detected with ECL reagent (Amersham Biosciences, Piscataway, NJ, USA).

### Construction of Histidine (His) tag and GST fusion protein

WASP15-His and GST-His fusion proteins have been described elsewhere [17]. cDNA fragments for Btk-SH3 were generated by PCR using a specific primer set (forward primer 5′-CGAATGCGGCCGCAATGACCGAGCTGAAAAAGGTC-3′ and reverse primer 5′-CGAATGCGGCCGCTCACTCATACATCTCTATGGAGTC-3′), and subcloned into the *Not*I site of the pGEX-4T-2 expression vector (GE Healthcare, Buckinghamshire, England). The GST-Btk-SH3 fusion protein was produced in BL21 *E. coli* cells and purified using a glutathione-sepharose 4B affinity chromatography column (GE Healthcare).

### His and GST pull-down assay

BMDMs were cultured in medium in either the presence or the absence of LPS (10 µg/mL) for 5 h, and then lysed with RIPA buffer (50 mM Tris-HCl buffer pH 7.6, 150 mM NaCl, 1% Nonidet P40, 0.5% Sodium Deoxy Cholate, Protease Inhibitor Cocktail; Nacalai Tesque, Kyoto, Japan) for 1 h at 4°C. The lysates were centrifuged at 10,000 × *g* for 10 min at 4°C, and the supernatants were incubated with GST-His or WASP15-His fusion proteins at 4°C overnight. The protein complexes were obtained using His Tagged Protein PURIFICATION KIT (MBL, Nagoya, Japan) according to the manufacturer's instructions, lysed with SDS sample buffer, and immunoblotted with anti-Btk polyclonal Ab (pAb) or anti-Grb2 pAb (Santa Cruz Biotechnology, CA, USA).

In the GST pull-down assay, the cleared lysates were incubated with glutathione sepharose (GE Healthcare) for 1 h at 4°C to remove nonspecifically bound proteins. The cleared lysates were incubated with glutathione sepharose beads bound to GST fusion protein at 4°C overnight. Beads were washed with PBS, lysed with SDS-sample buffer, and immunoblotted with anti-WASP mAb (which recognizes the WASP N-terminal domain) [39] or anti-GST pAb (MBL).

### Immunoprecipitation

BMDMs were lysed with RIPA buffer for 1 h at 4°C. The lysates were centrifuged at 10,000 × *g* for 10 min at 4°C, and incubated with Precleaning Matrix C (Santa Cruz Biotechnology) for 1 h at 4°C to remove nonspecifically bound proteins. The cleared lysates were incubated with anti-Btk mAb or anti-WIP pAb (Santa Cruz Biotechnology), and pulled down with Exacta Cruz C IP-matrix beads (Santa Cruz Biotechnology). After five washes with PBS, immunocomplexes were resuspended in SDS-sample buffer and boiled. The immunocomplexes were immunoblotted with anti-WASP pAb (raised against a synthetic peptide representing WASP residues 224–238, Upstate, Lake Placid, NY, USA), anti-T7 tag pAb (MBL), anti-Btk pAb, or anti-WIP pAb (Santa Cruz Biotechnology).

### Tyrosine phosphorylation of WASP, Btk, and Toll/IL-1 receptor domain-containing adapter protein (TIRAP)

BMDMs were cultured at 37°C in medium containing LPS (10 µg/mL) for 15 min, and then lysed with RIPA buffer (Nacalai Tesque) with Phosphatase Inhibitor Cocktail Set (Calbiochem, Darmstadt, Germany) for 1 h at 4°C. The lysates were centrifuged at 10,000 × *g* for 10 min at 4°C. The cleared lysates were incubated and pulled down with agarose-conjugated anti-phosphotyrosine (p-Tyr) mAb (Santa Cruz Biotechnology). After five washes with PBS, immunocomplexes were resuspended in SDS-sample buffer and boiled. The immunocomplexes were immunoblotted with anti-WASP pAb (Upstate, Lake Placid, NY, USA), anti-Btk pAb (Santa Cruz Biotechnology), or anti-TIRAP pAb (Abcam, Cambridge, UK).

### Ethics Statement

Procedures involving animal subjects have been approved by the Institutional Animal Care and Use Committee at the National Institute of Agrobiological Sciences (approval ID: H19-001-1).

## References

[1] Derry JM, Ochs HD, Francke U (1994). Isolation of a novel gene mutation in Wiskott-Aldrich syndrome.. Cell.

[2] Thrasher AJ (2002). WASP in immune-system organization and function.. Nat Rev Immunol..

[3] Cannon JL, Burkhardt JK (2004). Differential roles for Wiskott-Aldrich syndrome protein in immune synapse formation and IL-2 production.. J Immunol..

[4] Dupre L, Aiuti A, Trifari S, Martino S, Saracco P (2002). Wiskott-Aldrich syndrome protein regulates lipid raft dynamics during immunological synapse formation.. Immunity.

[5] Lorenzi R, Brickell PM, Katz DR, Kinnon C, Thrasher AJ (2000). Wiskott-Aldrich syndrome protein is necessary for efficient IgG-mediated phagocytosis.. Blood.

[6] Badolato R, Sozzani S, Malacarne F, Bresciani S, Fiorini M (1998). Monocytes from Wiskott-Aldrich patients display reduced chemotaxis and lack of cell polarization in response to monocyte chemoattractant protein-1 and formyl-methionyl-leucyl phenylalanine.. J Immunol..

[7] Zicha D, Allen WE, Brickell PM, Kinnon C, Dinn GA (1998). Chemotaxis of macrophages is abolished in the Wiskott-Aldrich syndrome.. Br J Haematol..

[8] Klm AS, Kakalls LT, Abdul-Manan N, Llu GA, Rosen MK (2000). Autoinhibition and activation mechamisms of the Wiskott-Aldrich syndrome protein.. Nature.

[9] Bouma G, Burns SO, Thrasher AJ (2009). Wiskott-Aldrich syndrome: Immunodeficiency resulting from defective cell migration and impaired immunostimulatory activation.. Immunobiology.

[10] Jin Y, Mazza C, Christie JR, Giliani S, Fiorini M (2004). Mutations of the Wiskott-Aldrich Syndrome protein (WASP): hotspots, effect on transcription, and translation and phenotype/genotype correlation.. Blood.

[11] Sato M, Tsuji NM, Gotoh H, Yamashita K, Hashimoto K (2001). Overexpression of the Wiskott-Aldrich syndrome protein N-terminal domain in transgenic mice inhibits T cell proliferative response via TCR signaling without affecting cytoskeletal rearrangements.. J Immunol..

[12] Sato M, Ogihara K, Sawahata R, Sekikawa K, Kitani H (2007). Impaired LPS-induced signaling in microglia overexpressing the Wiskott-Aldrich syndrome protein N-terminal domain.. Int Immunol.

[13] Akira S, Takeda K, Kaisyo T (2001). Toll-like receptors: critical proteins linking innate and acquired immunity.. Nat Immunol.

[14] Palsson-Mcdermott EM, O'neill LAJ (2004). Signal transduction by the lipopolysaccharide receptor, Toll-like receptor-4.. Immunology.

[15] Bogdan C, Rollinghoff M, Diefenbach A (2000). The role of nitric oxide in innate immunity.. Immunol Rev.

[16] Bogdan C (2001). Nitric oxide and the immune response.. Nat Immunol.

[17] Sato M, Sawahata R, Takenouchi T, Kitani H (2011). Identification of Fyn as the binding partner for the WASP N-terminal domain in T cells.. Int Immunol..

[18] Jefferies CA, Doyle S, Brunner C, Dunne A, Brint E (2003). Bruton's tyrosine kinase is a Toll/Interleukin-1 receptor domain-binding protein that participates in nuclear factor κB activation by Toll-like receptor 4.. J Biol Chem..

[19] Gray P, Dunne A, Brikos C, Jefferies CA, Doyle SL (2006). MyD88 adapter-like (Mal) is phosphorylated by Bruton's tyrosine kinase during TLR2 and TLR4 signal transduction.. J Biol Chem..

[20] de la Fuente MA, Sasahara Y, Calamito M, Anton IM, Elkhal A (2007). WIP is a chaperone for Wiskott-Aldrich syndrome protein (WASP).. Proc Natl Acad Sci USA.

[21] Ramesh N, Geha R (2008). Recent advances in the biology of WASP and WIP.. Immunol Res.

[22] Anton IM, Jones GE, Wandosell F, Geha R, Ramesh N (2007). WASP-interacting protein (WIP): working in polymerisation and much more.. Trends Cell Biol.

[23] Sheedy FJ, O'Neill LAJ (2007). The Troll in Toll: Mal and Tram as bridges for TLR2 and TLR4 signaling.. J Leukoc Biol..

[24] O'Neill LAJ, Bowie AG (2007). The family of five: TIR-domain-containing adaptors in Toll-like receptor signaling.. Nat Rev Immunol..

[25] Buss H, Dorrie A, Schmitz ML, Hoffmann E, Resch K (2004). Constitutive and Interleukin-1-inducible phosphorylation of p65 NF-κB at serine 536 is mediated by multiple protein kinases including IκB kinase (IKK)-α, IKKβ, IKKε, TRAF family member-associated (TANK)-binding kinase 1 (TBK1), and an unknown kinase and couples p65 to TATA-binding protein-associated factor II31-mediated interleukin-8 transcription.. J Biol Chem..

[26] Yang F, Tang E, Guan K, Wang C (2003). IKKβ plays an essential role in the phosphorylation of RelA/p65 on serine 536 induced by lipopolysaccharide.. J Immunol..

[27] Doyle SL, Jefferies CA, O'Neill LAJ (2005). Bruton's tyrosine kinase is involved in p65-mediated transactivation and phosphorylation of p65 on Serine 536 during NFκB activation by lipopolysaccharide.. J Biol Chem.

[28] Horwood NJ, Page TH, McDaid JP, Palmer CD, Campbell J (2006). Bruton's tyrosine kinase is required for TLR2 and TLR4-induced TNF, but not IL-6, production.. J Immunol..

[29] Mukhopadhyay S, Mohanty M, Mangla A, George A, Bal V (2002). Macrophage effector functions controlled by Bruton's tyrosine kinase are more crucial than the cytokine balance of T cell responses for microfilarial clearance.. J Immunol..

[30] Mohamed AJ, Yu L, Backesjo C, Vargas L, Faryal R (2009). Bruton's tyrosine kinase (Btk): function, regulation, and transformation with special emphasis on the PH domain.. Immunol Rev.

[31] Lindval JM, Blomberg KEM, Valiaho J, Vargas L, Heinonen JE (2005). Bruton's tyrosine kinase: cell biology, sequence conservation, mutation spectrum, siRNA modification, and expression profiling.. Immunol Rev..

[32] Cory GOC, Lovering RC, Hinshelwood S, MacCarthy-Morrogh L, Levinsky RJ (1995). The protein product of the c-*cbl* protooncogene is phosphorylated after B cell receptor stimulation and binds the SH3 domain of Bruton's tyrosine kinase.. J Exp Med.

[33] Guinamard R, Aspenstrom P, Fougereau M, Chavrier P, Guillemot J (1998). Tyrosine phosphorylation of the Wiskott-Aldrich syndrome protein by Lyn and Btk is regulated by CDC42.. FEBS Let.

[34] Matsushita M, Yamadori T, Kato S, Takemoto Y, Inazawa J (1998). Identification and characterization of a novel SH3-domain binding protein, Sab, which preferentially associates with Bruton's tyrosine kinase (Btk).. Biochem Biophys Res Commun.

[35] Cory GOC, MacCarthy-Morrogh L, Banin S, Gout I, Brickell PM (1996). Evidence that the Wiskott-Aldrich syndrome protein may be involved in lymphoid cell signaling pathways.. J Immunol.

[36] Bunnell SC, Henry PA, Kolluri R, Kirchhausen T, Rickles RJ (1996). Identification of Itk/Tsk Src homology 3 domain ligands.. J Biol Chem..

[37] Ramesh N, Anton IN, Hartwig JH, Geha RS (1997). WIP, a protein associated with Wiskott-Aldrich syndrome protein, induces actin polymerization and redistribution in lymphoid cells.. Proc Natl Acad Sci USA.

[38] Li X, Udagawa N, Takami M, Sato N, Kobayashi Y (2003). p38 Mitogen-activated protein kinase is crucially involved in osteoclast differentiation but not in cytokine production, phagocytosis, or dendritic cell differentiation of bone marrow macrophages.. Endocrinology.

[39] Sato M, Iwaya R, Ogihara K, Sawahata R, Kitani H (2005). Intrabodies against the EVH1 domain of Wiskott-Aldrich syndrome protein inhibit T cell receptor signaling in transgenic mice T cells.. FEBS J.

